# THE VOLUNTEER EXPERIENCE IN THE FAITH CARE FAMILY PROJECT FOR AFRICAN AMERICAN CAREGIVERS OF PERSONS WITH DEMENTIA

**DOI:** 10.1093/geroni/igad104.3669

**Published:** 2023-12-21

**Authors:** Noelle Fields, Ling Xu, Ishan Williams, Fayron Epps

**Affiliations:** The University of Texas at Arlington, Arlington, Texas, United States; The University of Texas at Arlington, Arlington, Texas, United States; University of Virginia, Charlottesville, Virginia, United States; Emory University, Atlanta, Georgia, United States

## Abstract

African American adults are disproportionately affected by Alzheimer’s disease and related dementias (ADRD). Improving knowledge about ADRD in the African American community is also important for dispelling the myths and stigma about the disease. The Faith Care Family (FCF) Project was developed to help bridge gaps in ADRD knowledge by training volunteers from a predominantly African American church (N = 9) to work with ADRD caregivers in a 6-week, telephone-based pilot intervention. The Alzheimer’s Disease Knowledge Scale (ADKS) and qualitative interviews were used to examine the church volunteers’ knowledge of dementia. Non-parametric McNemar tests and Wilcoxon signed-rank tests were conducted for each item and the sum scores of the ADKS scale, respectively. Qualitative interview data were analyzed using thematic analysis. The results of the quantitative data indicated overall significant improvements in the sum scores of ADKS after the training (p = .008) as well as after the intervention (p = .05). Some specific ADKS items showed a significant increase after the training including items related to high cholesterol, using reminder notes, and mental exercise as well as after the intervention related to prescription medication. Thematic analysis of the interviews revealed six themes: types of ADRD, ADRD symptoms/behaviors of ADRD, early-onset, fatality, dignity, and previous knowledge. Overall findings suggest that the FCF Project is a promising intervention for enhancing knowledge about ADRD for African American church volunteers. Recommendations for future research and strategies for lay provider ADRD interventions in faith communities are offered.

